# Impact of Tusk Anomalies on the Long‐Term Foraging Ecology of Narwhals

**DOI:** 10.1002/ece3.72376

**Published:** 2025-11-05

**Authors:** Marie Louis, Alba Rey‐Iglesia, Jennifer Routledge, Deon de Jager, Mikkel Skovrind, Mads Peter Heide‐Jørgensen, Thomas M. Kaiser, Kit M. Kovacs, Christian Lydersen, Aqqalu Rosing‐Asvid, Paul Szpak, Eline D. Lorenzen

**Affiliations:** ^1^ Greenland Institute of Natural Resources Nuuk Greenland; ^2^ Globe Institute, University of Copenhagen Denmark; ^3^ Department of Anthropology Trent University Peterborough Ontario Canada; ^4^ Centre for Taxonomy and Morphology, Section Mammalogy & Paleoanthropology Leibniz Institute for the Analysis of Biodiversity Change (LIB) Hamburg Germany; ^5^ Norwegian Polar Institute, Fram Centre Tromsø Norway

**Keywords:** cetaceans, dentition, genetic sexing, stable isotopes, *δ* ^13^C and *δ* ^15^N

## Abstract

Male narwhals are unique in having a long, spiralled tusk, while females of the species do not have a tusk. However, a small number of individuals develop tusk anomalies, including two‐tusked males or females with a tusk. In this study, we combine genetic sexing and bone collagen stable isotope (*δ* ^13^C and *δ* ^15^N) analysis to evaluate whether these tooth anomalies impact long‐term foraging ecology. Our analysis of individuals collected in the waters around Greenland showed no systematic impacts; eight of nine two‐tusked male narwhals and all three one‐tusked female narwhals fell within the normal range of known isotopic diversity from their source geographic regions. Two specimens with other forms of unusual dentition both showed stable isotope values outside the range of narwhals, suggesting that their diets were different. Therefore, the most common tusk anomalies in narwhals appear to have limited ecological consequences, while rarer forms of dentition are likely associated with altered foraging ecology.

## Graphical abstract

We combined stable δ^13^C and δ^15^N isotope analysis and genetic sexing to investigate whether narwhals with dental anomalies have a distinct long‐term foraging ecology. Our results showed no difference in stable isotope signature of two‐tusked male and one‐tusked female narwhals relative to normal‐tusked narwhals, indicating that an extra tusk does not impact narwhal foraging ecology. Two specimens with other odd dentition both showed stable isotope values far outside the range of narwhals, suggesting their diet was different.

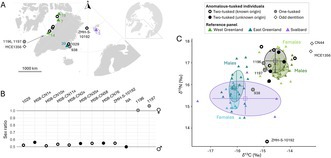

## 1 Introduction

The narwhal ( *Monodon monoceros* ) is found in the Atlantic sector of the High Arctic. Males are known for their iconic spiralled tusk, which is an erupted left canine tooth that has incremental annual growth, resulting in a tusk that can reach up to 3 m in length. Males also have an embedded tusk in their right maxilla, and females have two embedded tusks in their upper maxillary bone. Neither sex has teeth in the lower jaw. The erupted tusk in males is believed to be a secondary sexual trait used to attract potential female partners or to dissuade potential male competitors (Graham et al. 2020). It may also serve a function in prey handling and capture (O'Corry‐Crowe et al. 2025).

**FIGURE 1:**
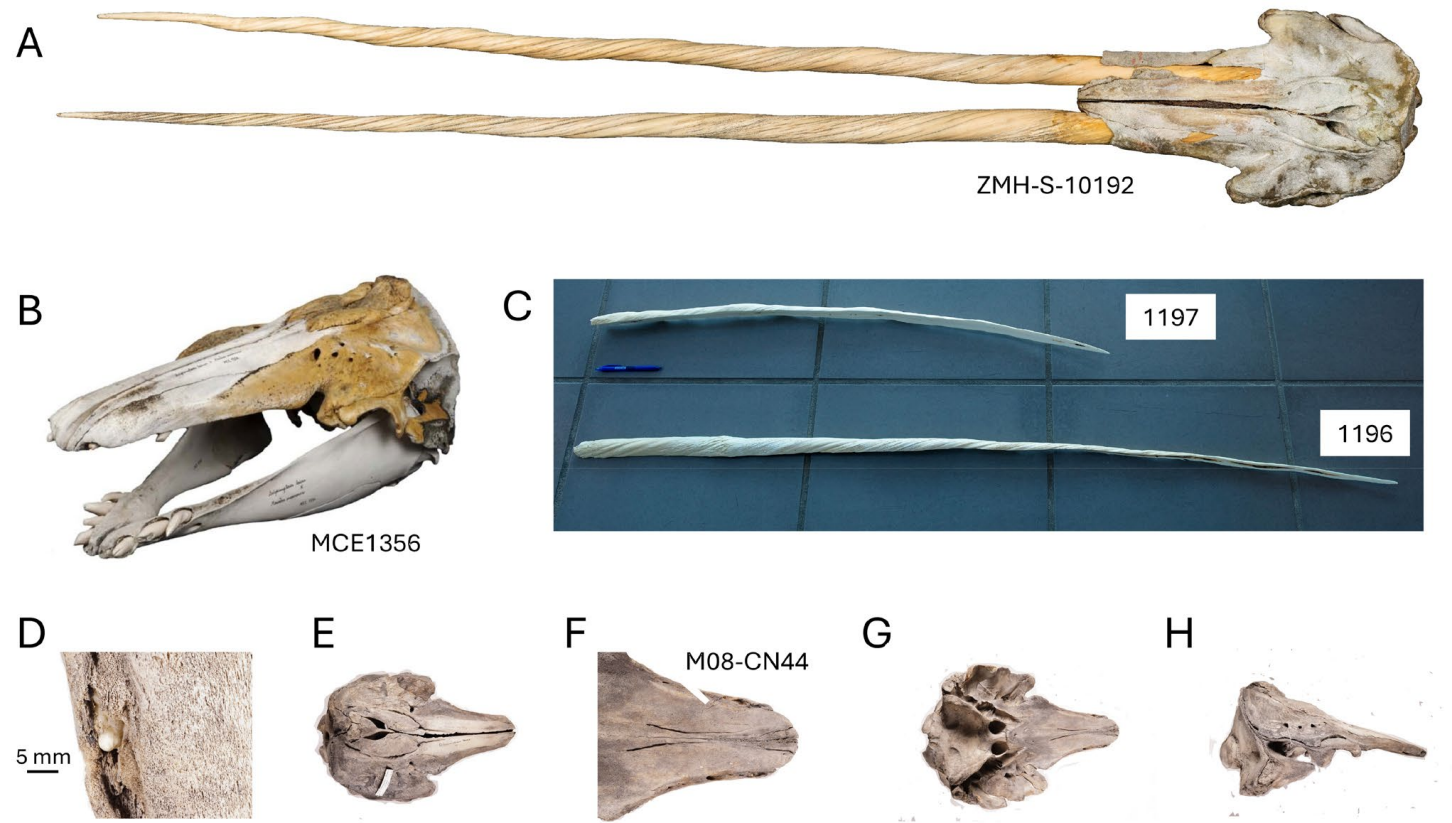
Images of five anomalous narwhal specimens. (A) Photograph of specimen ZMH‐S‐10192, courtesy of Mentz/Centrum für Naturkunde—Universität Hamburg. (B) Skull of specimen MCE1356, a first‐generation hybrid between a narwhal and a beluga, also known as Narluga, photo: Mikkel Høegh Post. (C) The single tusks from two putative females, specimens 1197 and 1196; both tusks have been sectioned, photo: Fernando Ugarte. (D–H) Images of specimen M08‐CN44, a narwhal with erupted teeth, photos: Mikkel Høegh Post.

Tusk anomalies in narwhals are known to occur at low frequency; in rare cases, the embedded tusk in males grows to a full‐length second tusk. Based on data from Greenland, this occurs in 0.9% of individuals. Males may also have no tusk (2.8%), and females can, on occasion, have one tusk (1.5%) (Garde and Heide‐Jørgensen 2022). Hay (1984) reported similar frequencies and types of narwhal tusk anomalies in Canada. There is one report of a two‐tusked female: an individual collected from the Greenland Sea in 1684 that is housed at Hamburg Universität, Germany (ZMH‐S‐10192), which was reportedly found in association with a foetus (Figure 1A, Figure S1) (Home 1813).

Tooth anomalies can lead to decreased body condition in mammals (Loe et al. 2006), or to shifts in foraging ecology, as was reported in an adult, first‐generation hybrid between a narwhal and a beluga ( *Delphinapterus leucas* ), with highly unusual dentition (Skovrind et al. 2019). This specimen has teeth in both the upper maxillary and the lower jaw, as in belugas (Figure 1B) (Heide‐Jørgensen and Reeves 1993). However, several of the teeth possess longitudinal grooves and are oriented horizontally in the same manner as a narwhal tusk (and embedded tusk(s)). Bone collagen stable *δ* ^13^C and *δ* ^15^N isotope analysis showed elevated *δ* ^13^C, indicating this hybrid animal (known colloquially as Narluga) had a distinct diet relative to either parental species (Skovrind et al. 2019). This observation motivated the present study to investigate whether tusk anomalies more broadly impact foraging ecology in narwhals.

Narwhals live most or all of the year in areas inaccessible to humans; they spend summers in High Arctic coastal waters and migrate offshore in the fall, and spend winters in the offshore pack ice, where much of their annual intake of food occurs (Chambault et al. 2023; Heide‐Jørgensen et al. 2013, 2015; Laidre and Heide‐Jørgensen 2005; Watt et al. 2015). For such animals, biochemical markers such as stable isotopes are useful tools to gain at least indirect knowledge about their foraging ecology (Watt et al. 2013; Louis et al. 2021). *δ* ^15^N provides information on the trophic level(s) at which an individual is feeding; there is a 3‰–5‰ increase in *δ* ^15^N in consumers relative to their prey (Minagawa and Wada 1984; Post 2002). *δ* ^13^C provides information on foraging habitat including pelagic versus demersal, or offshore versus coastal; pelagic and offshore organisms have lower *δ* ^13^C than demersal and coastal organisms (Peterson and Fry 1987; Newsome et al. 2010; France et al. 1998; Hobson et al. 1995; Clementz and Koch 2001). Due to the relatively slow turnover of bone collagen, stable isotope data represent the diet and foraging habitat of individuals over periods ranging from several years to decades (Wild et al. 2000; Hedges et al. 2007). Dentine forms in incremental layers, and thus the isotopic composition of different layers reflects different periods of an individual's life, depending on the species‐specific development of the tooth/tusk (Matthews and Ferguson 2014). In narwhals, as in other marine mammals, a growth layer in a tooth is assumed to represent a year (Hohn 2018).

Based on soft tissue (skin and muscle) *δ* ^13^C and *δ* ^15^N and on the analysis of stomach contents that provide direct data on prey consumed, narwhals feed on polar cod ( *Boreogadus saida* ), Arctic cod ( *Arctogadus glacialis* ), Greenland halibut ( *Reinhardtius hippoglossoides* ), capelin ( *Mallotus villosus* ) and squid ( *Gonatus fabricii* ), although the proportion of these different prey species varies by region (Baffin Bay, Northern Hudson Bay, East Greenland) (Watt et al. 2013; Laidre and Heide‐Jørgensen 2005; Garde et al. 2022).

In this study, we conduct genetic sexing and bone collagen stable isotope (*δ* ^13^C and *δ* ^15^N) analyses of a unique collection of 15 narwhals with rare tusk anomalies and analyse the data relative to 84 narwhals with normal dentition, to investigate potential impacts of dental anomalies on foraging ecology.

## 2 Materials and Methods

### 2.1 Specimens

Our study included genetic sexing and stable isotope (*δ* ^13^C and *δ* ^15^N) analyses of 15 anomalous‐tusked adult specimens (Table 1, Appendix, Table S1): ten two‐tusked individuals, three one‐tusked individuals believed to be females based on observations from hunters or biologists, Narluga (a first‐generation narwhal/beluga hybrid), and a narwhal with unusual dentition (Figure 1). We generated novel data for 12 of these specimens. Data for the other three specimens were sourced from the literature (Skovrind et al. 2019; Garde and Heide‐Jørgensen 2022; Rey‐Iglesia et al. 2022; Vicari et al. 2022) and were processed for the original publications in the same laboratory as the novel data. All 15 specimens are from the waters around Greenland and are housed in museums in Denmark and Germany, or at the Greenland Institute of Natural Resources (GINR).

**TABLE 1:** Metadata and provenance of the 15 anomalous‐tusked specimens analysed. Geographic region refers to West Greenland (WG), east of Greenland (EG), or Greenland (GL, if the specific region of origin is unknown).

Museum ID	Tusk anomaly	Locality	Region	Genetic sex	Substrate analysed (DNA/SIA)	Collection date	Institution
M08‐CN1x	Two‐tusked	NA	GL	M	tusk/bone	1800s	NHMD
M08‐CN10x	Two‐tusked	Itivdliarsuk	WG	M	tusk/bone	Oct 1883	NHMD
M08‐CN11x	Two‐tusked	Upernavik	WG	M	tusk/bone	May 1884	NHMD NHMD
M08‐CN35x	Two‐tusked	NA	GL	M	tusk/bone	1897	NHMD
M08‐CN58	Two‐tusked	Kap York	WG	M	tusk/bone	1921 (reg. date)	NHMD
M08‐CN76	Two‐tusked	NA	GL	M	tusk/bone	1965 (reg.date)	NHMD
M08‐CN2x	Two‐tusked	Uummannaq	WG	M	tusk/bone	1800s	NHMD
NA	Two‐tusked	NA	GL	M	tusk/bone	NA	Varde Museerne
ZMH‐S‐10192	Two‐tusked putative female	Greenland Sea	EG	M	tusk/bone	1684	LIB
1029	Two‐tusked	Scoresby Sound	EG	M	skin/NA	2009	GINR
1196	One‐tusked putative female	Kitsissuarsuit	WG	F	tusk/tusk	1996	GINR
1197	One‐tusked putative female	Kitsissuarsuit	WG	F	tusk/tusk	1996	GINR
938	One‐tusked female	Scoresby Sound	EG	NA	observation/tusk (Garde and Heide‐Jørgensen 2022)/(Rey‐Iglesia et al. 2022)	2017	NHMD/GINR
M08‐CN44	Odd dentition/unusual teeth (named CN44 in main text)	NA	NA	M	bone/bone (Vicari et al. 2022)/(Vicari et al. 2022)	1963	NHMD
MCE1356	Odd dentition/unusual teeth (Narluga)	Kitsissuarsuit	WG	M	bone/bone (Skovrind et al. 2019)/(Skovrind et al. 2019)	1986	NHMD

*Note:* The substrate used for DNA and stable isotope analysis (SIA) is indicated; dentine collagen was analysed from tusks and bone collagen from bone. Reg. date corresponds to registration date. Institution indicates where the specimen is housed: Natural History Museum of Denmark, University of Copenhagen (NHMD); Varde Museum; Greenland Institute of Natural Resources (GINR); Museum der Natur Hamburg of the Leibniz Institute for the Analysis of Biodiversity Change (LIB).

**FIGURE 2:**
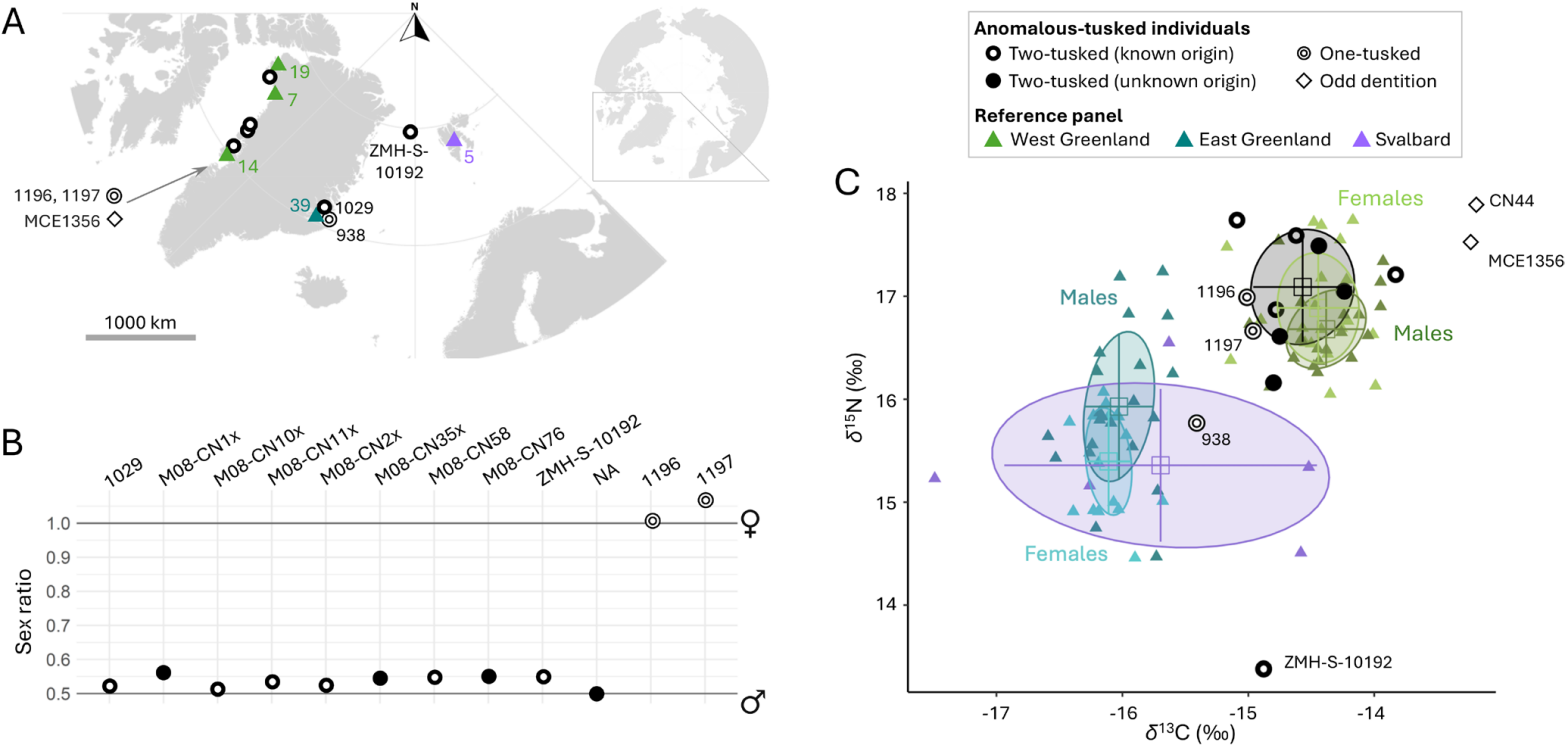
Samples and biomolecular results. (A) Map showing the sampling localities of the ten anomalous‐tusked individuals with known geographic origin and the 84 narwhals used as a reference panel for the stable isotope analysis. (B) Genetic sexing of ten two‐tusked (circles: known origin; dots: unknown origin) and two one‐tusked individuals. Genetic sex was determined by estimating the X chromosome:autosome coverage ratio (X:A ratio); values of ~0.5 indicate the individual is a male, and ~1.0 a female. (C) Bone collagen *δ* ^13^C and *δ* ^15^N of the 14 anomalous‐tusked individuals analysed and the reference panel split by geography and sex (West Greenland: 19 males, 20 females, one unknown sex; East Greenland: 22 males, 16 females, one unknown sex). The five Svalbard individuals (purple) were not genetically sexed. Shaded ovals indicate Bayesian standard ellipse areas for the individual groups (SEA_B_). The SEA_B_ of the two‐tusked individuals was estimated based on the eight individuals that were sampled in or assigned to West Greenland. Mean (square) and SD (error bars) are shown. Specific anomalous‐tusked individuals discussed in the text are indicated.

We generated novel DNA data for ten two‐tusked specimens and stable isotope data for nine of those specimens. For one individual (specimen 1029), we only had skin tissue and no bone; thus, it was only genetically sexed (Table 1). Of the nine two‐tusked individuals analysed with stable isotopes, four were from West Greenland. We assumed specimen M08‐CN10x (collected in October 1883), was from West Greenland, as the locality name in the museum ledger was ‘Itivdliarsuk’, which is the old name for Itilliarsuk, located in the Upernavik area. However, there is some uncertainty, as itivdliarsuk is also a Greenlandic name used to describe a landscape feature. Specimen M08‐CN11x was collected in Upernavik by the same person (Kolonibestyrer Ellberg) in May 1884, which further supports our assumption that M08‐CN10x is from West Greenland. Four two‐tusked individuals were known only to originate from ‘Greenland’, and thus we did not know their region of origin a priori. The remaining of the nine individuals was collected in the Greenland Sea (specimen ZMH‐S‐1019) between East Greenland and Svalbard (Figure 2A). This specimen has been presumed to be a female based on a contemporary news bulletin (Home 1813, Figure S1).

We also generated novel DNA and stable isotope data from two one‐tusked individuals, described as females by the hunters who harvested them. As mentioned above, females with tusks are rare (1.5%). Both of these tusks have an unusual shape and colour (Figure 1C). The tusks were part of a batch of eight tusks—four identified as coming from females by hunters—purchased in September 1996 by the general store (Kongelige Grønlandske Handel) in Kitsissuarsuit, West Greenland and brought to Nuuk. Kitsissuarsuit is the settlement where Narluga, the narwhal‐beluga hybrid, was collected (Heide‐Jørgensen and Reeves 1993). The Chairman of the hunter's organisation in Kitsissuarsuit was contacted by Aqqalu Rosing‐Asvid (GINR) for further information; he mentioned that several females with tusks had been caught that year and that tusked females had been caught previously around Kitsissuarsuit, which is not the case in other areas. The tusk from specimen 1197 is relatively short (115 cm) but has a clearly defined root (the part of the tusk that is embedded in the skull, Figure 1C). Normally, a 115 cm tusk is hollow for most of its length because the animal would be young and the tusk still growing, but this tusk was filled solidly with dentine and was about 0.5 kg (40%) heavier than a normal tusk of the same length. The tusk of specimen 1196 was longer (172 cm); it also had a defined root and was unusually slim. Another unusual feature was the reddish colour of the tusk, which may originate from algal pigments. Two of the other eight tusks were also relatively short (160 cm, 172 cm), also with roots, but they were not available for sampling.

In this study, we also included available stable isotope data from a third one‐tusked female (specimen 938), which was identified as female by biologists (Rey‐Iglesia et al. 2022), and from two individuals with highly unusual dentition, both genetically identified as male: specimen MCE1356 (Skovrind et al. 2019), known as Narluga, and specimen M08‐CN44 (named CN44 hereafter, Vicari et al. 2022). The latter had a cranial shape similar to narwhals, yet had a row with several erupted teeth in the upper maxillary (Figure 1D–H) (Vicari et al. 2022).

As a reference panel, we included bone collagen stable isotope *δ* ^13^C and *δ* ^15^N data from 84 adult or sub‐adult narwhals (Figure 2; Appendix, Figure S2): data from 40 individuals (19 males, 20 females, one unknown sex) from West Greenland and data from 39 individuals (22 males, 16 females, one unknown sex) from East Greenland (Louis et al. 2021). Five individuals from Svalbard were also included, from which novel stable isotope data were generated to expand the geographic range of the reference material (Appendix, Table S1). The Svalbard specimens were not genetically sexed.

### 2.2 Laboratory Analyses

#### 2.2.1 Genetic Sexing

We drilled 50–70 mg of dentine powder from nine two‐tusked narwhals and two putative one‐tusked females (Table 1). We carried out DNA extractions following a silica column‐based protocol with the binding buffer from Allentoft et al. (2015) (Westbury, Rey‐Iglesia, Cabrera, et al. 2025). We incubated dentine powder overnight at 37°C with constant rotation in 1 mL of extraction buffer. After the overnight incubation, any undigested powder was pelleted by centrifugation at 13,000 rpm in a microcentrifuge. In order to concentrate the DNA and reduce the sample volume for the following steps, the supernatant (~1 mL) was added to a 30 KDa Amicon Ultra‐4 filter and centrifuged at 4000 rpm until the volume reached 70 μL. The concentrated supernatant was combined with a 10× modified Qiagen PB buffer (i.e., 700 μL) (Allentoft et al. 2015), which was added to a Monarch spin column (NEB) and centrifuged for 1 min at 8000 rpm to bind the DNA to the silica membrane. The membrane‐bound DNA was washed twice with 650 μL Qiagen PE buffer by centrifugation at 5000 rpm for 1 min.

Prior to the library build, DNA extracts were treated with Thermolabile USER II enzyme (NEB). For each sample, the USER reaction was performed in 16 μL, with 2.4 μL of the Thermolabile USER II enzyme and 13.6 μL of each extract with an incubation time of 3 h at 37°C. USER‐treated DNA extracts were purified using Monarch columns (NEB) (Rey‐Iglesia, de Jager, Li, Cabrera, et al. 2025).

DNA extracts were transformed into single‐stranded DNA (ssDNA) sequencing libraries as in Kapp et al. (2021) (Rey‐Iglesia, de Jager, Li, and Lorenzen 2025) and double‐indexed using KAPA HiFi HotStart Uracil+ ReadyMix (KAPA Biosystems) (Westbury, Rey‐Iglesia, de Jager, et al. 2025). The resulting indexed libraries were quantified on a Qubit dsDNA HS (Invitrogen) and quality checked in the Agilent Fragment Analyzer. Indexed libraries were shotgun sequenced on an Illumina NovaSeq 6000 with the 150 base pairs (bp) PE technology.

For the two‐tusked individual for which only soft tissue was available for analysis, we used the Qiagen DNA blood and tissue kit, using the guidelines from the manufacturer with minor modifications (increase of the Proteinase *K* volume to 40 μL, incubation time was extended to 24 h, and tissue was manually lysed with a pillar after the night of incubation). The DNA sample was sent to Novogene for its library build and was sequenced on an Illumina NovaSeq 6000 with the 150 bp PE technology.

#### 2.2.2 Stable Isotopes

We sampled 300 mg of bone powder from the skulls of nine two‐tusked narwhals, and tusk powder from the base of the tusk of two putative one‐tusked females covering several growth layers, as no bone was available for these specimens (Table 1).

We did lipid extraction for the powdered samples with three repetitions of 10 mL 2:1 chloroform/methanol (v/v) under sonication for 1 h. After removing the solvent, we dried the samples under normal atmospheric pressure for 24 h. We demineralized the samples in 10 mL of 0.5 M HCl for 1 h while agitating them by an orbital shaker. After demineralization, we rinsed the samples to neutrality with Type I water, and heated them at 75°C for 36 h in 0.01 M HCl to solubilise the collagen. The water‐soluble collagen was transferred to a 4 mL glass vial, frozen and lyophilized. Upon analysis, there was an observed correlation between *δ* ^13^C and C:N_atomic_, indicating the presence of some residual lipids. To extract these lipids, we solubilised the lyophilized collagen and added chloroform and methanol to obtain a 10 mL solution of 10:5:4 chloroform/methanol/water (v/v/v) under sonication for 1 h. After centrifugation, the layer containing solubilised collagen and some methanol was collected and transferred to a new 4 mL glass vial; any residual methanol was evaporated from the solution at 60°C for 24 h. We freeze dried and weighed the samples before encapsulating 0.5 mg in tin for elemental and isotopic analysis.

We determined carbon and nitrogen stable isotopic and elemental compositions using a Euro EA 3000 Elemental Analyzer (Euro Vector SpA) coupled to a Nu Horizon (Nu Instruments, UK) continuous flow isotope ratio mass spectrometer at the Water Quality Centre at Trent University, Canada. We calibrated stable carbon and nitrogen isotopic compositions relative to the VPDB and AIR scales using USGS40 and USGS41a (glutamic acid) (Qi et al. 2016, 2003). Analytical uncertainty was monitored using four internal reference materials in addition to USGS40 and USGS41a: SRM‐1 (caribou bone collagen, long‐term average *δ* ^13^C = −19.38‰ ± 0.10‰, *δ* ^15^N = +1.86‰ ± 0.15‰), SRM‐2 (walrus bone collagen, long‐term average *δ* ^13^C = −14.78‰ ± 0.06‰, *δ* ^15^N = +15.57‰ ± 0.20‰), SRM‐14 (polar bear bone collagen, long‐term average *δ* ^13^C = −13.61‰ ± 0.08‰, *δ* ^15^N = +21.52‰ ± 0.17‰) and SRM‐17 (phenylalanine, long‐term average *δ* ^13^C = −12.41‰ ± 0.10‰, *δ* ^15^N = +3.18‰ ± 0.22‰). For samples analysed in duplicate, the mean difference between pairs was 0.06‰ for *δ* ^13^C and 0.11‰ for *δ* ^15^N. Standard uncertainty was determined to be ±0.11‰ for *δ* ^13^C and ±0.21‰ for *δ* ^15^N (Szpak et al. 2017).

We adjusted the *δ* ^13^C values to correct for the change in atmospheric and oceanic dissolved inorganic carbon that has occurred since the late 19th century due to industrialisation (the “Suess Effect”; (Keeling et al. 1979; Quay et al. 1992)), following (Szpak, Julien, Royle, Savelle, et al. 2020), although we used 0.014 for the annual rate at which *δ* ^13^C has declined for a particular water body (Mellon 2018). The formula used is as follows:

$$\delta 13C\;\text{Suess}=0.014\times \left(e{\left(\text{collection year}-1850\right)}^{0.027}\right)+\delta 13C$$

We did not correct *δ* ^13^C values for the Suess effect for specimens for which the exact collection date is unknown (but they are known to have originated in the 1800s, according to the museum's records), because this phenomenon had no effect prior to around 1850. We used the registration date for specimens for which collection date was not available (Table 1). For atmospheric CO_2_ the shift between 1850 and 1930 is 0.3‰ (more likely 0.1‰ in the ocean), and thus a collection date earlier than the entry date would have little effect on our results.

To analyse bone and dentine (tusk) data together, dentine isotopic values need to be translated into bone‐equivalents using a correction factor, and we thus applied a correction factor to the *δ* ^13^C and *δ* ^15^N values (0.81 and 0.49 were subtracted, respectively) obtained from the two tusks for which we did not have accompanying bone (specimens 1196 and 1197) (Rey‐Iglesia et al. 2022).

### 2.3 Data Analyses

#### 2.3.1 Genetic Sexing

We used the SeXY pipeline to identify the sex of our sampled individuals (Cabrera et al. 2022). First, we identified scaffolds putatively originating from the sex chromosomes in the narwhal reference genome assembly (GCF_005190385.1). For this, we aligned the narwhal reference genome to the Cow X (Genbank accession: AC_000187.1) and Human Y (Genbank accession: NC_000024.10) sex chromosomes using satsuma synteny v.2.1 (Grabherr et al. 2010) with default parameters. Then, we mapped the raw reads to the narwhal reference assembly using paleomix v.1.3.8 (Schubert et al. 2014) with BWA v.0.7.15 backtrack algorithm (Li and Durbin 2009), requiring a minimum mapping quality of 30 and removing duplicates. We removed any risk of human contamination by mapping the narwhal fastq files to the human genome (hg38.UCSC.fasta) to generate bam files, only keeping reads not mapping to the human genome. We followed the SeXY pipeline (https://github.com/andreidae/SeXY) (Cabrera et al. 2022) and used the “Coverage_calculation.sh” script to estimate the average coverage of sequencing reads aligning to the X chromosome and the autosomes. We determined the sex of each specimen by estimating the X chromosome:autosome coverage ratio (X:A ratio). Specimens with X:A ratio < 0.7 were determined as males, and those with X:A ratio > 0.8 as females. Mapping statistics, mean ratios and coverage can be found in Table S2.

#### 2.3.2 Stable Isotopes

We ran all statistical analyses in R v.3.6.1 (R Core Team 2021). We compared the *δ* ^13^C and *δ* ^15^N of the two‐tusked narwhals with the reference data set for narwhals collected in the same region (West Greenland, *n* = 40) using Student's *t*‐tests. For this analysis, we included eight two‐tusked narwhals; four were known to originate from West Greenland, four grouped in the region based on preliminary *δ* ^13^C and *δ* ^15^N analysis (see Results below). The analysis did not include specimen ZMH‐S‐10192, which was sampled in the Greenland Sea between East Greenland and Svalbard. We also compared the same eight two‐tusked narwhals with the reference data for males and females separately (*n*_M_ = 19, *n*_F_ = 20, one individual did not have sex information) using Student's *t*‐tests. Our data satisfied the assumption of normality and homogeneity of variance for the different levels of subdivision.

For the three putative one‐tusked females and the two otherwise anomalous specimens Narluga and CN44, sample sizes were too low to run statistical analyses, and thus we evaluated the results visually.

Using isotopic niche as a proxy for ecological niche, we compared the isotopic niches of the West Greenland groups (two‐tusked vs. normal; vs. males; vs. females) using Bayesian multivariate ellipse‐based metrics implemented in the packages SIBER and rjags (Jackson et al. 2011; Bearhop et al. 2004; Newsome et al. 2007; Plummer et al. 2016). We calculated standard ellipse areas corrected for sample size (SEA_C_), and Bayesian standard ellipses (SEA_B_) for each group. We estimated SEA_B_ using 10^5^ posterior draws, a burnin of 10^3^ and a thinning of 10, and used SEA_B_ to test for differences in niche width among groups (i.e., the proportion (p) of draws of the posterior distribution of the SEA_B_ in which the area of one group was smaller than the other). We evaluated isotopic niche similarity between two groups as the proportion (%) of the non‐overlapping area of the maximum likelihood (ML) fitted ellipses of the two.

We also generated SEA_B_ for the five Svalbard individuals; the individuals were not sexed and were thus pooled. We did not run any statistics on this region due to low sample size. We also visually compared the one‐tusked females and the two individuals with highly unusual dentition (Narluga and CN44) with the reference panels from their region of origin.

## 3 Results

The ten two‐tusked individuals were all identified as male, with X:A ratios between 0.50 and 0.56 (Figure 2B, Appendix, Table S2). This included specimen ZMH‐S‐10192, which was a priori assumed to be a female (Figure S1). The two other putative one‐tusked females analysed were confirmed as such, with X:A ratios of 1.0 and 1.07.

We did not have specific locality information for all individuals analysed, and four of the two‐tusked individuals were known only to originate from ‘Greenland’ (Table 1). The isotopic niches of narwhals in West and East Greenland do not overlap (Louis et al. 2021) and have discrete carbon isotope values (range West: −15.17‰ to −13.93‰, range East: −16.59‰ to −15.60‰, after correcting for the Suess effect) (Louis et al. 2021). The four two‐tusked narwhals that were known a priori to originate from West Greenland grouped with the reference dataset from this region, including specimen M08‐CN10x, thus confirming our assumption that it originated from there (Figure 2C, Figure S2). The four specimens with only ‘Greenland’ as known provenance similarly grouped with the West Greenland narwhals (Figure 2C). We thus assigned these individuals to an origin in West Greenland and included all eight samples in the statistical analysis.

We did not find any significant difference in *δ* ^13^C and *δ* ^15^N between the two‐tusked individuals and the West Greenland reference panel (*t* = −1.09, df = 8.81, *p* = 0.30 for *δ* ^13^C; *t* = 1.45, df = 9.22, *p* = 0.18 for *δ* ^15^N, Figure S2). Similarly, there were no differences between the two‐tusked individuals and the reference data from West Greenland when split by sex: males (*t* = 1.20, df = 10.84, *p* = 0.26 for *δ* ^13^C; *t* = −1.98, df = 10.02, *p* = 0.08 for *δ* ^15^N); females (*t* = 0.80, df = 10.86, *p* = 0.44 for *δ* ^13^C; *t* = −0.91, df = 12.73, *p* = 0.38 for *δ* ^15^N, Figure 2C). The isotopic niche of the two‐tusked narwhals (SEA_B_ = 0.75‰^2^) was not significantly different (proportion *p* = 0.86) from the reference panel (SEA_B_ = 0.46‰^2^, Figure S2). Nor were there any significant differences found between the two‐tusked narwhals and the West Greenland males (*p* = 0.73, SEA_B_ = 0.55‰^2^) or females (*p* = 0.95, SEA_B_ = 0.63‰^2^, Figure 2C). Ellipse overlap between the two‐tusked narwhals and the reference panel isotopic niches was 35%, 20% with the males and 49% with the females.

Due to the absence of bone collagen *δ* ^13^C and *δ* ^15^N data from narwhals in Svalbard, we generated novel data from five individuals, including three stranded animals that were opportunistically sampled in the region (Appendix, Table S1). To ascertain if narwhals from East Greenland and from Svalbard are isotopically differentiated, we generated Bayesian standard ellipses for each of the three sampled reference populations (also incl. West Greenland, Figure 2C). Although based on a limited sample size, the range of *δ* ^15^N observed in Svalbard was within the range observed in East Greenland. The *δ* ^13^C values of the Svalbard individuals had a wide range, overlapping with both West and East Greenland, which may reflect some of the samples being derived from stranded specimens, and thus perhaps originating from elsewhere. The isotopically derived ecological niche of the Svalbard individuals encompassed most of the niche of East Greenland narwhals (Figure 2C).

The bone collagen *δ* ^13^C value for specimen ZMH‐S‐10192 was within the range of values observed in narwhals from Svalbard (Figure 2C). However, the individual's *δ* ^15^N was much lower than any other sample included in our analysis. Visual inspection of the stable isotope values retrieved for the other anomalous‐tusked individuals, where sample size prevented statistical analyses, indicated the two West Greenland one‐tusked females had values within the range observed for the West Greenland female reference panel (Figure 2C). The one‐tusked female from East Greenland had *δ* ^15^N values within the range observed for the East Greenland female reference panel, although the *δ* ^13^C value of this individual was somewhat higher (by 0.27‰). Specimen CN44, the male narwhal with unusual teeth, had a higher *δ* ^13^C value than any other narwhal analysed, similar to Narluga (Skovrind et al. 2019).

## 4 Discussion

By combining genetic sexing and bone collagen stable isotope (*δ* ^13^C and *δ* ^15^N) analysis, we evaluate whether tusk anomalies in narwhals impact foraging ecology. Our stable isotope values are mostly from bone, which has a slow turnover, ranging from years to decades. Therefore, the isotopic compositions presented in this study reflect general, long‐term foraging ecology (Gage et al. 1989; Wild et al. 2000; Hedges et al. 2007).

We compared narwhals with tusk anomalies with a reference panel of narwhals from West and East Greenland (Louis et al. 2021), and from Svalbard.

Four two‐tusked individuals that were only known to originate in ‘Greenland’ were assigned to West Greenland based on their isotopic signature. When grouped with the four two‐tusked individuals with known West Greenland origin, we found no significant differences in their *δ* ^13^C or *δ* ^15^N values and the reference panel from the region (Figure S2). The findings suggest having two tusks does not impact isotopically derived resource use. We acknowledge that our sample size is low, and thus our findings should be interpreted with caution. We assumed isotopic stability across the 200 years covered by the West Greenland samples (Table 1), implying that there has been no significant shift in stable isotope values apart from the Suess effect, which we account for in our analyses. This is supported by a comparable bone collagen *δ* ^13^C and *δ* ^15^N analysis of belugas; belugas sampled from their summering ground in Elwin Bay, High Arctic Canada, between 1874 and 1898, and from their West Greenland wintering grounds in the 1990s show no significant shifts in *δ* ^13^C and *δ* ^15^N across time, with mean *δ* ^13^C = −13.4‰ and *δ* ^15^N = +17.4‰ in the late 19th (Szpak, Julien, and Royle 2020) and mean *δ* ^13^C = −13.66 and *δ* ^15^N = +17.35 in the 1990s (Skovrind et al. 2019), after correcting for the Suess effect (Szpak, Julien, Royle, Savelle, et al. 2020).

Isotopic differentiation has been observed between male and female narwhals in East Greenland, where males have higher *δ* ^15^N than females (Louis et al. 2021), but this is not the case in West Greenland. We wanted to test whether there were any differences between the two‐tusked individuals from West Greenland and each sex separately. We did not find significant differences in these comparisons. The size of the isotopic niche of the two‐tusked narwhals did not differ significantly from those of the reference panel, also when analysed by sex. The two‐tusked individuals appear to have a relatively large niche, despite the small sample size (*n* = 8), which might reflect that the two‐tusked individuals come from several and unknown localities in West Greenland and may represent different stocks, which use different wintering/summering grounds and migration routes between them (Heide‐Jørgensen et al. 2013; Hobbs et al. 2019). Alternatively, it might reflect individual variation in foraging ecology, which has been observed in narwhals using stable isotopes in dentine tusk layers (Zhao et al. 2022; Dietz et al. 2021).

The two‐tusked specimen ZMH‐S‐10192 was caught in the Greenland Sea, between East Greenland and Svalbard, in 1684 (Figures 1A and 2A, Figure S1). The specimen was assumed to be a female due to a contemporary news bulletin that describes a foetus found in association with the adult animal (Figure S1; Home 1813). However, our genetic sexing identified the individual as a male, as was the case for the other two‐tusked specimens investigated (Figure 2B). The isotopically derived foraging ecology of ZMH‐S‐10192 differed markedly from the reference panel from the region, with a δ^13^C value (−14.88‰) higher than East Greenland narwhals (mean = −16.06‰), but similar to two of the Svalbard individuals (with values of −14.56‰ and −14.58‰, Figure 2C). The δ^15^N value (+13.38‰) was 1.1‰ lower than the lowest values observed in East Greenland/Svalbard (+14.46‰ and +14.51‰, respectively), indicating ZMH‐S‐10192 was possibly feeding at a lower trophic level than narwhals in the region (mean East Greenland = +15.7‰, mean Svalbard = +15.34‰). The narwhals in the East Greenland reference panel were caught between 1993 and 2007 (Louis et al. 2021), and the Svalbard individuals (for which collection dates exist) were collected between 1886 and 2014 (Table 1). ZMH‐S‐10192 thus differs from the other two‐tusked narwhals by its much older age. However, no temporal stable isotope data are available from East Greenland from this period and therefore we cannot rule out that the lower *δ* ^15^N value may be due to ecological changes over time. Alternatively, it may be due to shifts in *δ* ^15^N at the base of the food web due to reconfiguration of the primary producers, which would affect values observed in top predators, even if the latter feed on the same prey or at the same trophic level (Sherwood et al. 2014). Thus, with the data available, we are unable to further elucidate why we observe a low *δ* ^15^N value in ZMH‐S‐10192.

We evaluated whether having a tusk in females has any impact on foraging ecology. The two one‐tusked females from West Greenland had isotopic values (specimen 1196, *δ* ^13^C: −15.01‰ and *δ* ^15^N: +16.99‰; specimen 1197, *δ* ^13^C: −14.96‰ and *δ* ^15^N: +16.66‰), which are within the range of the reference data from the region (*δ* ^13^C: −15.17‰ to −13.99‰ and *δ* ^15^N: +16.05‰ to +17.74‰, Figure 2C); the higher value of *δ* ^13^C (−15.41‰) in the one‐tusked female from East Greenland relative to the reference panel (range: −16.42‰ to −15.68‰) is within the analytical uncertainty of the method.

Overall, the lack of a systematic difference in the isotopically derived foraging ecology of anomalous‐tusked narwhals relative to normal‐tusked narwhals suggests the presence of an extra tusk does not impact narwhal foraging ecology significantly. Additionally, the one‐tusked female from East Greenland, which was a carcass examined by biologists, showed signs of multiple pregnancies (Garde and Heide‐Jørgensen 2022), indicating that the presence of a tusk does not interfere with female reproductive success.

We assessed whether other rare forms of tooth anomalies found in Narluga and specimen CN44—where individuals have multiple erupted teeth similar to belugas—could affect their diet. Narluga is from West Greenland (Skovrind et al. 2019), but the sampling locality of specimen CN44 is unknown (Vicari et al. 2022). The latter had elevated *δ* ^15^N and *δ* ^13^C, similar to values observed in the Narluga. The elevated *δ* ^13^C may indicate a more demersal foraging ecology (Hobson et al. 1995; Skovrind et al. 2019). It could also reflect the use of more coastal environments (Clementz and Koch 2001; Burton and Koch 1999). Although based on only two specimens, our findings suggest the presence of beluga‐like erupted teeth in narwhals does impact foraging ecology, possibly allowing individuals to capture or handle other types of prey compared to normal narwhals.

We cannot determine prey species based on our approach, but the elevated *δ* ^15^N values (+17.52‰ for Narluga and +17.89‰ for CN44) were in the top of the range observed in narwhals and close to values found in belugas in West Greenland (mean = +17.3‰ (Louis et al. 2021)). The diet of narwhals and belugas overlap; both species target Greenland halibut ( *R. hippoglossoides* ), Arctic cod ( *B. saida* ), polar cod ( *A. glacialis* ) and capelin ( *M. villosus* ) (Heide‐Jørgensen and Teilmann 1994; Laidre and Heide‐Jørgensen 2005; Marcoux et al. 2012; Watt et al. 2013; Watt and Ferguson 2015; Yurkowski et al. 2017). However, belugas consume less squid—which occupy lower trophic levels—than narwhals and also less Atlantic cod ( *Gadus morhua* ) and other fish, including redfish ( *Sebastes mentella* ) (Degerbøl and Nielsen 1930), which feed at higher trophic levels (Hansen et al. 2012). The presence of teeth may also allow belugas, Narluga and CN44 to feed on larger animals, which would have higher ^15^N values.

## 5 Conclusions

Based on bone and dentine collagen stable isotope (*δ* ^13^C and *δ* ^15^N) analysis, our findings suggest the vast majority (12 out of 13) of the two and one‐tusked anomalous individuals analysed are within the isotopic niche of normal‐tusked narwhals, and thus tusk anomalies do not appear to systematically impact foraging ecology. Although our sample size is small, the rarest type of tusk anomaly (presence of beluga‐like erupted teeth) does appear to impact foraging ecology. Our analysis also illustrates the potential of using stable isotopes to assign specimens to a geographic region of origin in narwhals, as we were able to do for the four two‐tusked individuals of unknown provenance.

## Author Contributions

**Marie Louis:** conceptualization (equal), formal analysis (lead), investigation (equal), visualization (lead), writing – original draft (lead). **Alba Rey‐Iglesia:** formal analysis (supporting), investigation (equal), writing – review and editing (equal). **Jennifer Routledge:** investigation (equal), writing – review and editing (equal). **Deon de Jager:** investigation (equal), writing – review and editing (equal). **Mikkel Skovrind:** investigation (equal), writing – review and editing (equal). **Mads Peter Heide‐Jørgensen:** data curation (equal), writing – review and editing (equal). **Thomas M. Kaiser:** data curation (equal), writing – review and editing (equal). **Kit M. Kovacs:** data curation (equal), writing – review and editing (equal). **Christian Lydersen:** data curation (equal), writing – review and editing (equal). **Aqqalu Rosing‐Asvid:** data curation (equal), writing – review and editing (equal). **Paul Szpak:** funding acquisition (equal), resources (equal), supervision (equal), writing – review and editing (equal). **Eline D. Lorenzen:** conceptualization (lead), funding acquisition (lead), resources (lead), supervision (lead), visualisation (lead), writing – original draft (equal).

## Conflicts of Interest

The authors declare no conflicts of interest.

## Supporting information


**Appendix S1:** ece372376‐sup‐0001‐AppendixS1.docx.

## Data Availability

Appendix, Table S1 for the stable isotope data. The raw sequencing files, input files and scripts are available at the Electronic Research Data Archive at the University of Copenhagen: https://sid.erda.dk/sharelink/dUlpjPmGbI.
