# Supplementary material for: Impact of Tusk Anomalies on the Long‐Term Foraging Ecology of Narwhals
Source: Ecol Evol. 2025 Nov 5;15(11):e72376. doi: 10.1002/ece3.72376 (PMC12588726; doi:10.1002/ece3.72376)
Supplement: Supplementary file 1 — Appendix S1: ece372376‐sup‐0001‐AppendixS1.docx. [file ECE3-15-e72376-s001.docx]

Appendix

Impact of tusk anomalies on the long-term foraging ecology of narwhals

Marie Louis^1,2^*, Alba Rey Iglesia^2^, Jennifer Routledge^3^, Deon de Jager^2^, Mikkel Skovrind^2^, Mads Peter Heide-Jørgensen^1^, Thomas M. Kaiser^4^, Kit M. Kovacs^5^, Christian Lydersen^5^, Aqqalu Rosing-Asvid^1^, Paul Szpak^3^, Eline D. Lorenzen^2^*

1. Greenland Institute of Natural Resources, Kivioq 2, 3900 Nuuk, Greenland
2. Globe Institute, University of Copenhagen, [Øster Voldgade 7, 1350 København, Denmark](https://www.google.com/maps/place//data=!4m2!3m1!1s0x4652530fa5b45383:0x99312bd074c7643f?sa=X&ved=1t:8290&ictx=111)
3. Department of Anthropology, Trent University, 1600 West Bank Drive, Peterborough, Ontario K9L 0G2, Canada
4. Centre for Taxonomy and Morphology, Section Mammalogy & Paleoanthropology, Leibniz Institute for the Analysis of Biodiversity Change (LIB), Martin-Luther-King-Platz 3, Hamburg 20146, Germany
5. Norwegian Polar Institute, Fram Centre, N-9296 Tromsø, Norway

*Corresponding authors: Marie Louis: marielouis17@hotmail.com and Eline Lorenzen: elinelorenzen@sund.ku.dk

ORCID: ML, 0000-0002-4611-5503; ARI, 0000-0002-3768-9185; JR, 0000 0002 1827 7767; DdJ, 0000-0003-4388-2624; MS, 0000-0002-5430-5884; MPH-J, 0000-0003-4846-7622; KMK, 0000-0002-5878-4819; ARA, 0000-0001-7184-4305; PS, 0000-0002-1364-6834; EDL, 0000-0002-6353-2819

Overview of the Appendix

figure S1

tables S1 and S2


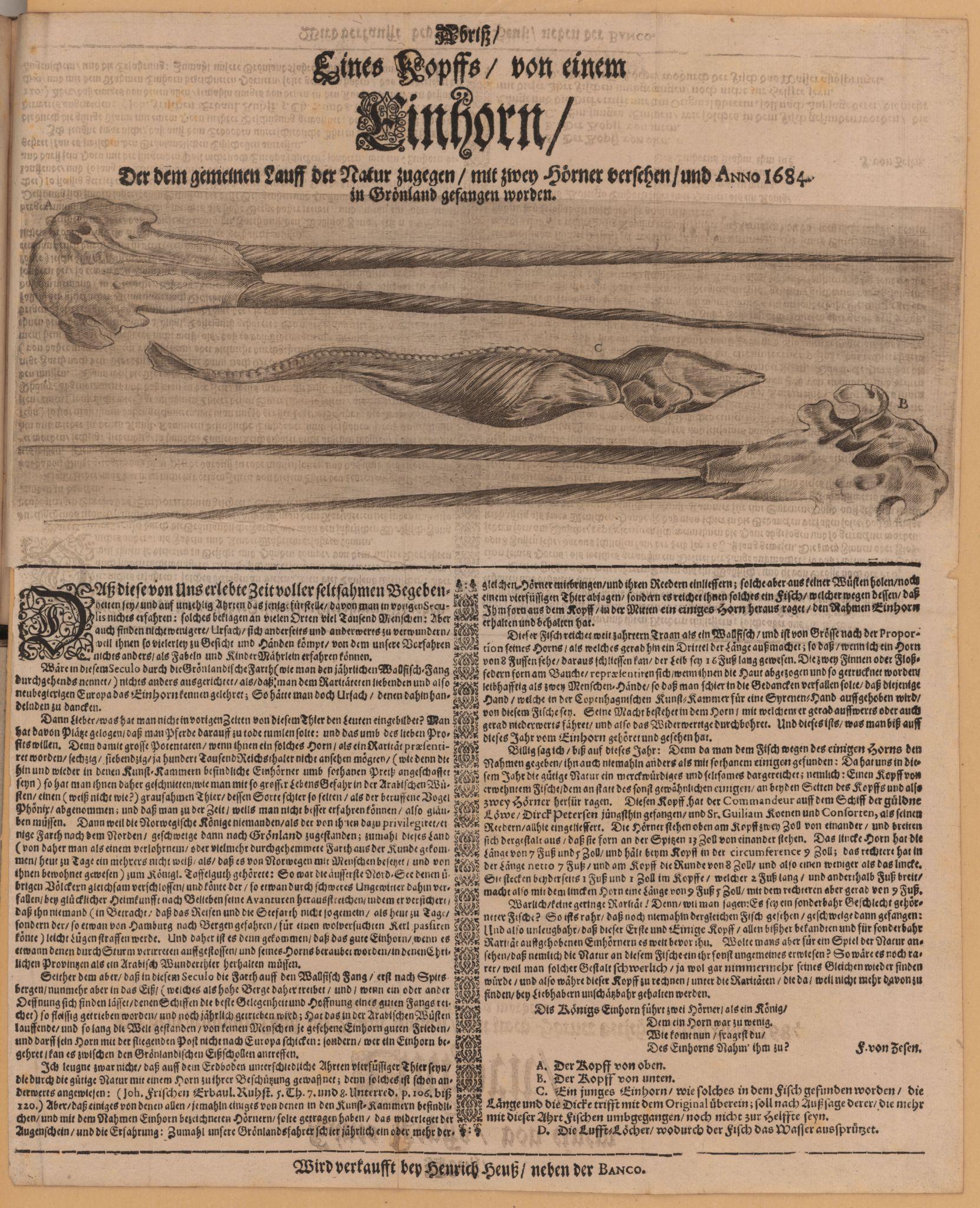


**Figure S1.** A broadsheet in German from 1684 detailing the find of a two-tusked female narwhal from the Greenland Sea (specimen ZMH-S-10192). The broadsheet text explains the origin of the *unicorn*. It praises the rarity and high trade value of the two-tusk specimen: *It is so rare / that no one has seen such a fish / let alone caught one*. The caption of the depicted foetus leaves room for interpretation: *A young unicorn / as such found in the fish / the length and thickness match the original*. The text thus avoids to explicitly state that the foetus depicted was taken from the two-tusked narwhal. The print is based on an original from the Carl-von-Ossietzky State and University Library, Hamburg.

**Figure S2. Bone collagen *δ*^13^C and *δ*^15^N of the 14 anomalous‐tusked individuals analysed and the reference panel split by geography** (West Greenland: 40 individuals; East Greenland: 39 individuals, Svalbard: 5 individuals). Shaded ovals indicate Bayesian standard ellipse areas for the individual groups (SEA_B_). The SEA_B_ of the two-tusked individuals was estimated based on the eight individuals that were sampled in or assigned to West Greenland. Mean (square) and SD (error bars) are shown. Specific anomalous-tusked individuals discussed in the text are indicated.

**Table S1. Sample and data overview.** Included are the 15 anomalous-tusked and five Svalbard individuals for which novel data were generated for this study. Institution indicates where the specimen is housed: Natural History Museum of Denmark, University of Copenhagen (NHMD); Varde Museum; Greenland Institute of Natural Resources (GINR); Museum der Natur Hamburg of the Leibniz Institute for the Analysis of Biodiversity Change (LIB); Norwegian Polar Institute (NPI); Natural History Museum, University of Oslo (NHM); Swedish Museum of Natural History (NMR). TEAL (Trent Environmental Archaeology Lab) ID indicates the sample ID of University of Trent, where the stable isotope data were generated. Stable isotope (SI) data include *δ*^13^C corrected for the Suess effect. Geographic region refers to West Greenland (WG), east of Greenland (EG), or Greenland (GL, if specific region of origin is unknown).

| **Institution** | **Institution**  **ID** | **TEAL**  **ID** | **Sex** | **SI** | ***δ*^13^C (‰)** | ***δ*^13^C Suess (‰)** | ***δ*^15^N (‰)** | **C/N** | **Year for Suess correction** | **Collection year** | **Registration year** | **Locality and region** | **Comments** |
| --- | --- | --- | --- | --- | --- | --- | --- | --- | --- | --- | --- | --- | --- |
| NHMD | M08-CN1x | 5295 | M | new | -14.8 | -14.8 | 16.16 | 3.23 | 1800 |  | 1800 | GL | specimen from the Royal Art Chamber, could be even older (as early as 1700s) |
| NHMD | M08-CN10x | 5296 | M | new | -14.62 | -14.58 | 17.59 | 3.46 | 1883 | 1883 | 1884 | Itivdliarsuk, WG |  |
| NHMD | M08-CN11x | 5297 | M | new | -14.78 | -14.74 | 16.87 | 3.41 | 1884 | 1884 | 1884 | Upernavik, WG |  |
| NHMD | M08-CN35x | 5298 | M | new | -14.49 | -14.44 | 17.49 | 3.56 | 1897 |  | 1897 | GL |  |
| NHMD | M08-CN58 | 5301 | M | new | -15.19 | -15.09 | 17.74 | 3.41 | 1921 |  | 1921 | Kap York, WG |  |
| NHMD | M08-CN76 | 5302 | M | new | -14.56 | -14.24 | 17.05 | 3.23 | 1965 |  | 1965 | GL | bought from a person in Copenhagen in 1965 |
| NHMD | M08-CN2x | 10501 | M | new | -13.83 | -13.83 | 17.21 | 3.32 | 1800 |  | 1800 | Omenakfjord, WG | specimen from the Royal Art Chamber, could be even older (as early as 1700s) |
| Varde Museerne | NA | 16393 | M | new | -14.75 | -14.75 | 16.61 | 3.4 | NA |  |  | Greenland |  |
| LIB | ZMH-S-10192 | 23849 | M | new | -14.88 | -14.88 | 13.38 | 3.24 | 1684 | 1684 |  | Greenland Sea (between Svalbard and EG) | SI run in duplicate with nearly same values found (δ13C: -14.92, δ15N: 13.28) |
| GINR | 1029 | NA | M | NA | NA | NA | NA | NA | 2009 | 2009 |  | Scoresby Sound, EG |  |
| GINR | 1196 | 17466 | F | new | -15.73 | -15.01 | 16.99 | 3.13 | 1996 | 1996 |  | Kitsissuarsuit, WG | correction factor dentine to bone applied |
| GINR | 1197 | 17467 | F | new | -15.68 | -14.96 | 16.66 | 3.15 | 1996 | 1996 |  | Kitsissuarsuit, WG | correction factor dentine to bone applied |
| NHMD/GINR | 938 | 11295 | F  [(Garde and Heide-Jørgensen 2022)](https://paperpile.com/c/QurilJ/tlXfK) | [(Rey-Iglesia et al. 2022)](https://paperpile.com/c/QurilJ/zogbe) | -16.68 | -15.41 | 15.77 | 3.18 | 2017 | 2017 |  | Scoresby Sound, EG |  |
| NHMD | M08-CN44 | 604 | M  [(Vicari et al. 2022)](https://paperpile.com/c/QurilJ/FKMeH) | [(Vicari et al. 2022)](https://paperpile.com/c/QurilJ/FKMeH) | -13.52 | -13.22 | 17.89 | 3.19 | 1963 | NA | 1963 | GL |  |
| NHMD | MCE1356 | 575 | M  [(Skovrind et al. 2019)](https://paperpile.com/c/QurilJ/Ugd0b) | [(Skovrind et al. 2019)](https://paperpile.com/c/QurilJ/Ugd0b) | -13.84 | -13.23 | 17.52 | 3.2 | 1986 | 1986 |  | Kitsissuarsuit, WG | Also named Narluga |
| NPI | M.sv.4/SV08 | 17468 | NA | new | -16.26 | -16.26 | 15.16 | 3.1 | NA |  |  | Svalbard | sampled in 2012, also named CGG-1-012748 |
| NPI | MM2014/01 | 17469 | M | new | -16.8 | -15.63 | 16.55 | 3.14 | 2014 | 2014 |  | Recherchefjorden, Svalbard | subadult male skeleton found onshore, also named CGG-1-017632 |
| NHMO | NHMO-DMA-46574/1-O | 14143 | NA | new | -17.54 | -17.49 | 15.23 | 3.53 | 1900 |  |  | Svalbard | uncertainty about date, also named CGG-1-024518 |
| NRM | NRM-MA558407 | 14545 | NA | new | -14.56 | -14.52 | 15.34 | 3.19 | 1886 | 1886 |  | Svalbard |  |
| Svalbard Museum | SVB 7773 | 17902 | NA | new | -14.58 | -14.58 | 14.51 | 3.14 | NA |  | 2014 | Svalbard | fragment of tusk, correction factor dentine to bone applied |

**Table S2. Mapping statistics and genetic sexing.** The number of unique reads corresponds to the number of remaining mapped reads after taking out the PCR duplicates. Genetic sex was determined by estimating the X chromosome:autosome (A) coverage ratio (X:A ratio).

| **Specimen** | **Nb of mapped reads** | **Nb of unique reads** | **Mean coverage X** | **Mean coverage A** | **Mean X:A ratio** |
| --- | --- | --- | --- | --- | --- |
| M08-CN1x | 2882876 | 2524340 | 0.03 | 0.05 | 0.56 |
| M08-CN10x | 720006 | 620759 | 0.01 | 0.02 | 0.52 |
| M08-CN11x | 1091075 | 966276 | 0.02 | 0.03 | 0.54 |
| M08-CN35x | 2599245 | 2270864 | 0.03 | 0.05 | 0.55 |
| M08-CN58 | 4584063 | 4050866 | 0.06 | 0.1 | 0.55 |
| M08-CN76 | 2471885 | 2182721 | 0.04 | 0.07 | 0.55 |
| M08-CN2x | 4931349 | 4334837 | 0.06 | 0.12 | 0.53 |
| NA | 1057809 | 902396 | 0.02 | 0.04 | 0.5 |
| ZMH-S-10192 | 6061129 | 5924311 | 0.08 | 0.14 | 0.55 |
| 1029 | 76477141 | 60425812 | 2.05 | 3.9 | 0.53 |
| 1196 | 6265412 | 5770557 | 0.24 | 0.24 | 1.01 |
| 1197 | 4151302 | 3837687 | 0.13 | 0.12 | 1.07 |

**Literature cited**

[Garde, Eva, and Mads Peter Heide-Jørgensen. 2022. “Tusk Anomalies in Narwhals (Monodon Monoceros) from Greenland.” *Polar Research* 41 (June). https://doi.org/](http://paperpile.com/b/QurilJ/tlXfK)[10.33265/polar.v41.8343](http://dx.doi.org/10.33265/polar.v41.8343)[.](http://paperpile.com/b/QurilJ/tlXfK)

[Rey-Iglesia, Alba, Tess Wilson, Jennifer Routledge, Mikkel Skrovind, Eva Garde, Mads Peter Heide-Jørgensen, Paul Szpak, and Eline D. Lorenzen. 2022. “Combining δ13C and δ15N from Bone and Dentine in Marine Mammal Palaeoecological Research: Insights from Toothed Whales.” *Isotopes in Environmental and Health Studies*. 59. https://doi.org/](http://paperpile.com/b/QurilJ/zogbe)[10.1080/10256016.2022.2145285](http://dx.doi.org/10.1080/10256016.2022.2145285)[.](http://paperpile.com/b/QurilJ/zogbe)

[Skovrind, Mikkel, Jose Alfredo Samaniego Castruita, James Haile, Eve C. Treadaway, Shyam Gopalakrishnan, Michael V. Westbury, Mads Peter Heide-Jørgensen, Paul Szpak, and Eline D. Lorenzen. 2019. “Hybridization between Two High Arctic Cetaceans Confirmed by Genomic Analysis.” *Scientific Reports* 9 (1): 7729.](http://paperpile.com/b/QurilJ/Ugd0b)

[Vicari, Deborah, Eline D. Lorenzen, Mikkel Skovrind, Paul Szpak, Marie Louis, Morten T. Olsen, Richard P. Brown, et al. 2022. “Skull Ecomorphological Variation of Narwhals (Monodon Monoceros, Linnaeus 1758) and Belugas (Delphinapterus Leucas, Pallas 1776) Reveals Phenotype of Their Hybrids.” *PloS One* 17 (8): e0273122.](http://paperpile.com/b/QurilJ/FKMeH)
